# Meta-lens light-sheet fluorescence microscopy for *in vivo* imaging

**DOI:** 10.1515/nanoph-2021-0748

**Published:** 2022-02-21

**Authors:** Yuan Luo, Ming Lun Tseng, Sunil Vyas, Ting-Yu Hsieh, Jui-Ching Wu, Shang-Yang Chen, Hsiao-Fang Peng, Vin-Cent Su, Tzu-Ting Huang, Hsin Yu Kuo, Cheng Hung Chu, Mu Ku Chen, Jia-Wern Chen, Yu-Chun Chen, Kuang-Yuh Huang, Chieh-Hsiung Kuan, Xu Shi, Hiroaki Misawa, Din Ping Tsai

**Affiliations:** National Taiwan University, Institute of Medical Device and Imaging, No. 1 Ren Ai Rd. Sect. 1, Taipei, 10051, Taiwan, ROC; Institute of Electronics, National Yang Ming Chiao Tung University, Hsinchu, Taiwan, ROC; Department of Clinical Laboratory Sciences and Medical Biotechnology, National Taiwan University, No. 1., Chang-Te St., Taipei, 100, Taiwan, ROC; Electrical Engineering, National United University, No. 2, Lienda, Miaoli, 36003, Taiwan, ROC; Mechanical Engineering, National Taiwan University, No. 1, Sec. 4, Roosevelt Rd., Taipei, 10617, Taiwan, ROC; Department of Electrical Engineering, City University of Hong Kong, Kowloon, Hong Kong; Hokkaido University, Sapporo, Hokkaido, Japan; Department of Electrical Engineering, The Hong Kong Polytechnic University, City University of Hong Kong, Kowloon, Hong Kong

**Keywords:** fluorescence microscopy, light sheet microscopy, metasurface

## Abstract

Light-sheet fluorescent microscopy has become the leading technique for *in vivo* imaging in the fields of disease, medicine, and cell biology research. However, designing proper illumination for high image resolution and optical sectioning is challenging. Another issue is geometric constraints arising from the multiple bulky components for illumination and detection. Here, we demonstrate that those issues can be well addressed by integrating nanophotonic meta-lens as the illumination component for LSFM. The meta-lens is composed of 800-nm-thick GaN nanostructures and is designed for a light-sheet well-adapted to biological specimens such as the nematode *Caenorhabditis elegans* (*C. elegans*). With the meta-lens, the complexity of the LSFM system is significantly reduced, and it is capable of performing multicolor fluorescent imaging of live *C. elegans* with cellular resolution. Considering the miniature size and plane geometry of the meta-lens, our system enables a new design for LSFM to acquire *in vivo* images of biological specimens with high resolution.

## 1 Introduction

**Figure 1:**
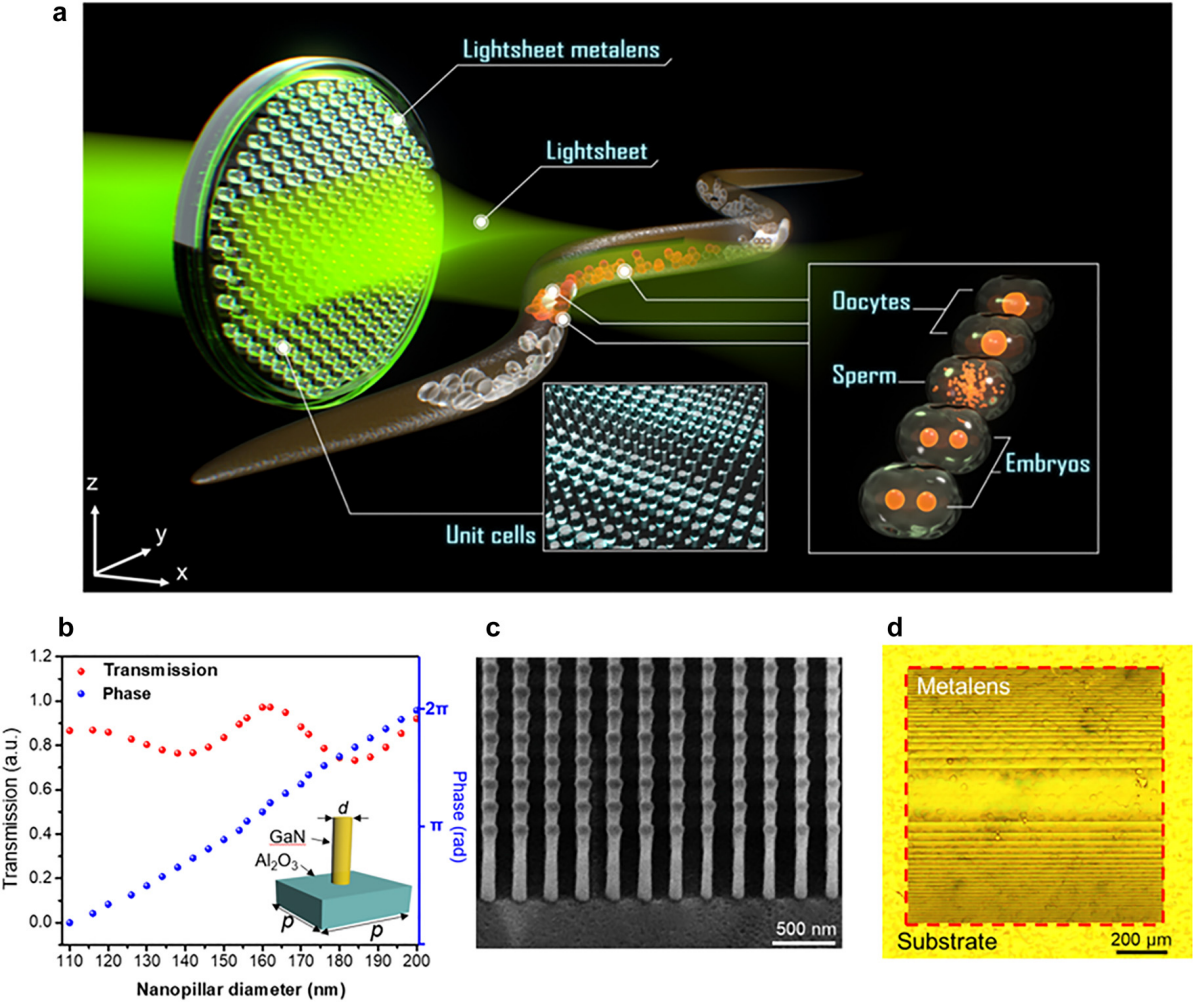
Meta-lens for light-sheet fluorescence microscopy (LSFM). (a) Schematic of the LSFM for fluorescence imaging of a *C. elegans*. The meta-lens consists of 800-nm dielectric nanopillars for locally modulating the phase. Inset shows the schematic diagram of fluorescent images of oocytes, sperms, and embryos in the *C. elegans*. (b) The simulation results of the phase modulations (blue points) and the transmissions (red points) of the nanopillars with different diameters. Inset: the geometric parameters of the nanopillars. *d*: diameter. *p*: period of the unit cell, 300 nm. The height of the nanopillar is 800 nm. (c) Titled-viewed SEM image of nanopillars in the light-sheet meta-lens. (d) An optical microscope image of the light-sheet meta-lens.

Fluorescence imaging of fine structures in live specimens provides a powerful way to understand cellular and subcellular dynamics in biology and clinical applications. Among current microscopic imaging systems, lightsheet fluorescent microscopy (LSFM) [1], [2], [3], [4], [5], [6] has become the leading technique for this purpose in recent years. In the measurement using an LSFM [1], [2], [3], the specimen is generally illuminated side-on using a thin sheet of light, with beam waist smaller than feature sizes of a target specimen, to offer optical sectioning capability in a high-speed fashion. Fluorescence images from the illuminated section can then be observed along the detection axis, which is orthogonal to the light-sheet excitation plane. Due to the unique orthogonal scheme between excitation and collection, LSFM has multiple advantages, including a large field of view (FoV), high image resolution, and low photo-damage [3, 4, 11, 12]. This efficient imaging technique has led to numerous cutting-edge findings and helped solve many problems in various fields [4, 12], [13], [14]. However, the unique experimental configuration of LSFM also results in many challenges. Constructing advanced imaging systems usually suffers from the requirements of bulky optical components, and this issue is even more serious for building a miniature LSFM system [15], [16], [17]. It is because the cumbersome excitation/imaging optical components, as well as the sample holder, need to be integrated together in a very limited space. This seriously restricts the space for placing and tracing bio-specimens as well. A promising route to effectively solve this problem is to introduce metasurface photonics [7], [8], [9], [10, 18], [19], [20], [21], [22], [23], [24], [25], [26], [27] in the system. Metasurfaces consist of arrays of subwavelength photonic resonators as unit cells that can efficiently modulate amplitude/phase. With its capability to control light in the nanoscale, metasurface optics provides a great platform for realizing planar photonic devices with functionalities on demand [7, 20], [21], [22, 28], [29], [30], [31], [32], [33], [34], [35], [36], [37]. Here, we demonstrate that the LSFM system complexity can be reduced substantially by integrating a light-sheet metasurface lens (called light-sheet meta-lens) for imaging live *Caenorhabditis elegans*, as depicted in Figure 1a. We developed an ultrathin meta-lens to significantly shrink the size of the illumination arm in an LSFM from the tens of centimeter-scale into the operation wavelength scale. We also demonstrate that, with a well-crafted nanophotonic meta-lens, a light-sheet microscope can have much compact size and have a comparable imaging capability as its conventional counterpart.

## 2 Results and discussion

### 2.1 Design and fabrication of the nanophotonic meta-lens

As illustrated in Figure 1, the light-sheet meta-lens is composed of lithography-defined GaN nanopillars as subwavelength resonators, which can locally modulate the optical phase. To demonstrate the imaging capability of the LSFM system equipped with proposed light-sheet meta-lens, fluorescent imaging of cellular structures in live *C. elegans* was performed (Figure 1a). *C. elegans* is an important model organism in the study of human diseases [38, 39], drug discovery [40], and developmental biology [41]. In addition to a short life cycle and simple body plan, the transparent body allows direct observation of subcellular dynamics with fluorescence tags in live *C. elegans* animals. However, due to the large length-to-width ratio (can be larger than 200), proper illumination for creating optical sections on the whole animal is challenging. As a result, though light-sheet fluorescent microscopy has been successfully used to trace rapid embryo development [42] and the movement of the whole animal [43, 44], high-resolution *in vivo* imaging of subcellular dynamics in live *C. elegans* using compact LSFM systems can be demanding. Therefore, a well-designed light-sheet is essential to achieve good optical sectioning as well as low out-of-focus noise. Another issue is associated with the difficulty of realizing an LSFM system capable of efficiently fixing, identifying, and tracking tiny *C. elegans*. Due to the great degree of freedom of metasurface optics for designing the optical functionalities at the nanoscale, producing a meta-lens with light-sheet parameters well-adapted to the parameters of a *C. elegans* is feasible. Also, the ultra-compact size of the light-sheet meta-lens offers flexibility for the design of a system with a proper space to track and fix small *C. elegans*. In this way, the meta-lens enables a proper illumination condition to image a live *C. elegans*. A fully-grown *C. elegans* is generally around 1-mm long and 50-μm wide. The structures of interest, such as the oocytes and the embryos, are of a few tens of micrometers. Therefore, to provide a suitable illumination condition, the length, beam waist, and FoV of the meta-lens are designed to be 1 mm, 6 µm, and 105 µm, respectively.

To realize the proposed light-sheet meta-lens with a nanoscale thickness, designing a group of GaN nanopillars that can cover a full 2*π* phase range is the key issue. Commercial FDTD software CST was used to simulate the properties of 800-nm GaN nanopillars with different diameters. Under illumination, waveguide-like modes induced in the nanopillars can effectively modulate the phase of the output light (supplementary information Figure S1). A full 2*π* phase modulation range (the blue data points in Figure 1b) with high transmission (>73%, the red data points in Figure 1b) of the nanopillars is successfully achieved via changing the nanopillars’ diameter. Notably, different from the unit cells used in our previous designs [8, 9, 34], the nanopillars are circularly symmetric, and thus they are not sensitive to polarization states under normal illumination. The polarization-independent property of the nanopillars ensures that the light-sheet meta-lens does not require any additional polarization components. In the design of the light-sheet meta-lens, the phase profile was numerically calculated (supplementary information Figure S2). The meta-lens contains more than 10 million nanopillars with different diameters are arranged along the substrate surface according to the phase profile. The resulting light-sheet meta-lens is 1 mm in length and was fabricated using electron-beam lithography followed by an etching process on an 800-nm GaN film (see supplementary information) [33]. A scanning electron microscope (SEM) image of the GaN nanopillars in the light-sheet meta-lens and an optical microscope image of the light-sheet meta-lens are shown in Figure 1c and d, respectively.

### 2.2 LSFM system characterization

**Figure 2:**
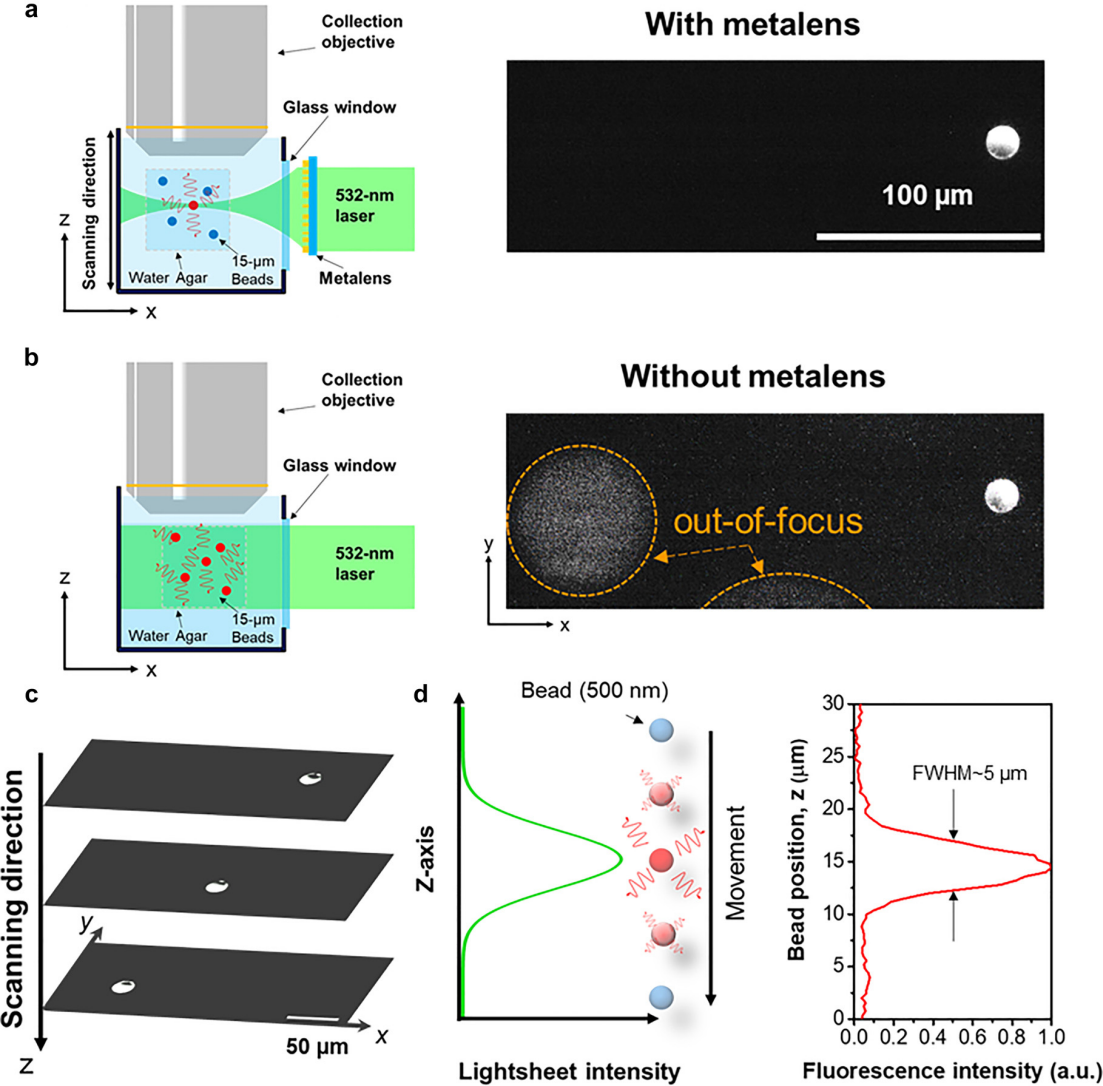
Characterization of the meta-lens-equipped LSFM system. Comparison of imaging performance using 15-µm fluorescent beads (a) with and (b) without the meta-lens. Left panels in (a) and (b) are schematic diagrams of the setup, and the corresponding fluorescence images are presented in the right panels. (c) Lightsheet scanning along the *z*-axis (with the meta-lens), three individual beads can be observed at three different layers, without any obvious out-of-focus background. (d) Optical sectioning capability test by using a 0.5-µm fluorescent bead. Left: a schematic diagram of the measurement. Right: the corresponding fluorescent intensity profile at different positions.

The imaging capability of our meta-lens-equipped LSFM for volumetric samples is tested by using fluorescent beads embedded in agar. Fluorescent beads are widely utilized to measure system performance as well as optical sectioning capability. In our experimental measurement, a 532-nm laser was used for excitation. A comparison of fluorescence images taken with and without the light-sheet meta-lens is presented in Figure 2a and b, respectively. With the light-sheet generated by the meta-lens, only a single bead (diameter: 15 µm, Fluoresbrite® YG Microspheres, Polysciences) was excited and observed in Figure 2a. Conversely, after removing the meta-lens, out-of-focus background noise from de-focused beads was observed (highlighted by orange circles in Figure 2b), indicating poor optical sectioning capability without light-sheet illumination. To demonstrate the 3D scanning capability of the system, the sample holder was scanning in the axial direction (*i.e.*, the *z*-axis), and fluorescence images of beads at different depths were recorded accordingly. The images of the fluorescent beads, located at different positions, and the associated video can be found in Supplementary video 1. As shown in Figure 2c, while scanning the light-sheet along the *z*-axis, three individual fluorescent beads can be seen at different layers without any out-of-focus background. Furthermore, a 0.5-µm fluorescent bead in agar was used to measure the optical sectioning capability (axial resolution) of our LSFM system by gradually scanning along the *z*-axis. The schematic diagram and experimental measurement of fluorescence intensity along axial scanning are shown in Figure 2d. A Gaussian-like intensity distribution is observed in the plot, and the full width at half maximum (FWHM) of the curve is ∼5 µm. Therefore, we summarize the system shows the essential advantages of general LSFM systems, and it can be used for imaging fluorescence specimens.

### 2.3 In vivo imaging of *C. elegans*

**Figure 3:**
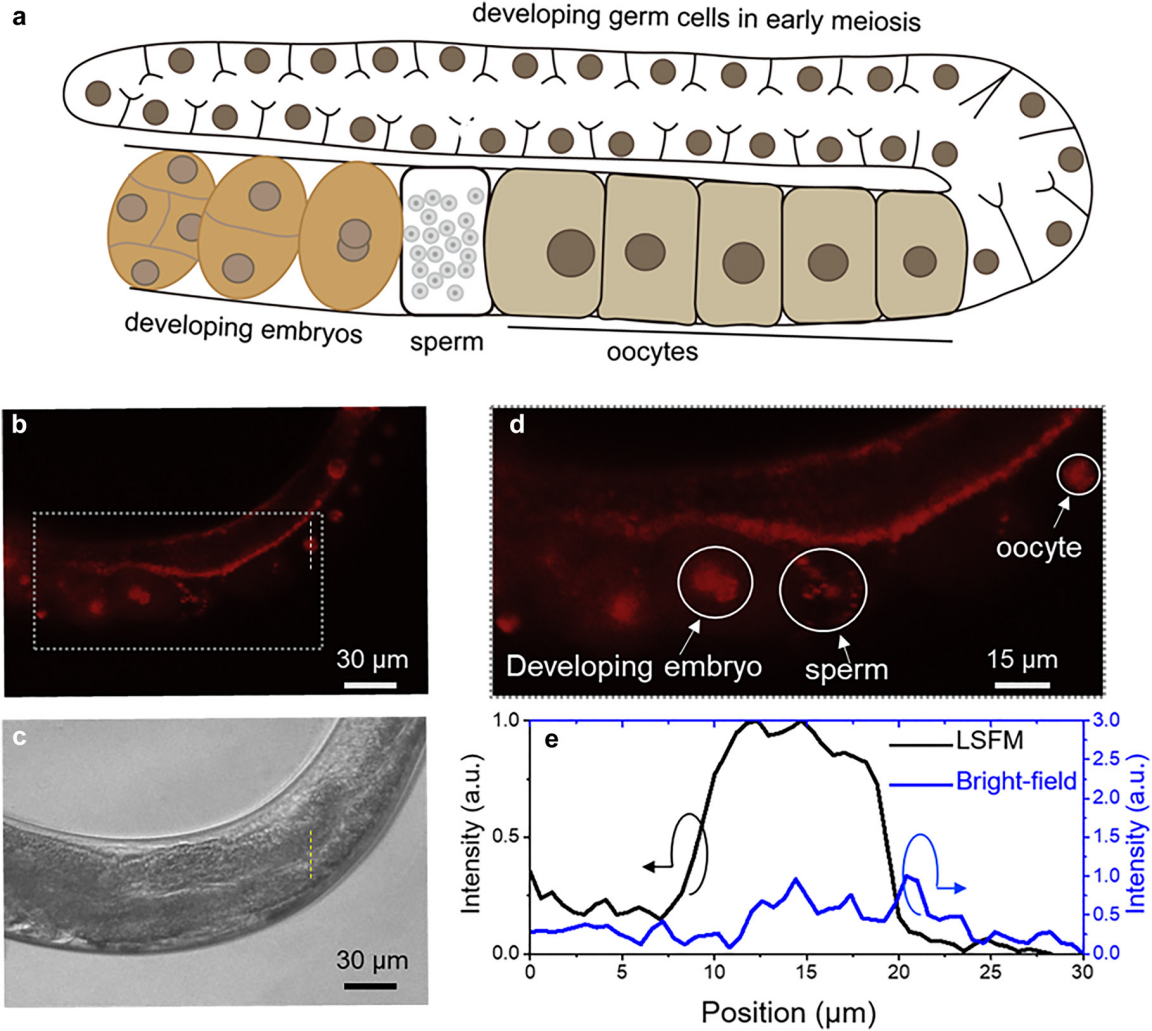
*In vivo* fluorescence images of mCherry-tagged oocysts and embryos in a *C. elegans*. (a) Schematic diagram of a *C. elegans*. (b) LSFM image of the *C. elegans* immobilized in agar. (c) Bright-field image of the *C. elegans*. (d) The corresponding zoom-in LSFM image. (e) Intensity cross-sections along the lines plotted in (b) and (c), respectively. (b) and (c) are shown on the same scale.

**Figure 4:**
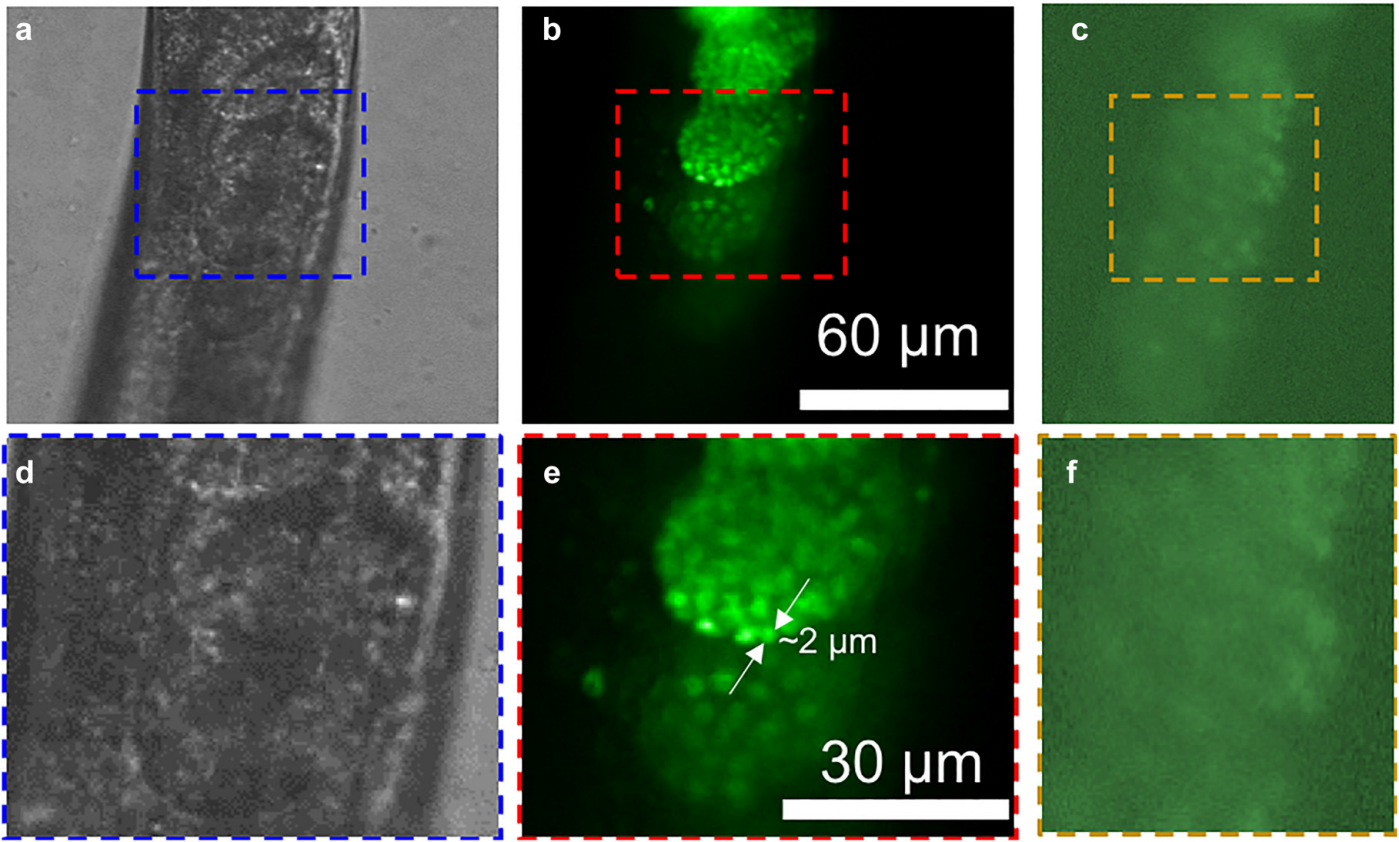
*In vivo observation of green fluorescence from GFP in a C. elegans.* To image the green fluorescence emitted from the fluorescently tagged *C. elegans*, a 491-nm laser is utilized as the excitation of our LSFM. The peak wavelength of the fluorescence is at 517 nm. (a) Bright-field image of the *C. elegans* immobilized in agar. (b) LSFM image of the *C. elegans*. (c) Wide-field fluorescence image of the *C. elegans*. (d)–(f) Zoom-in to the dashed-box regions shown in (a)–(c), respectively. (d)–(f) are shown in the same scale.

To demonstrate the capability of the system for fluorescence imaging of live animals at the cellular level, we demonstrate the observation of the developing process by examination of the germline of live *C. elegans*. The *C. elegans* germline exhibits a series of germ cell nuclei at different development stages, as depicted in Figure 3a. Anesthesia immobilized *C. elegans* were mounted into a slab of agar and transferred under the meta-lens-equipped LSFM setup for observation. Detailed worm manipulation procedures can be found in the Methods section. In order to examine the performance of our LSFM system for imaging live *C. elegans*, under different fluorescence, mCherry [45] or green fluorescent protein (GFP) [44] tagged histone was used to label germ cell nuclei, as presented in Figures 3 and 4. In the experiment, the excitation wavelength for the mCherry and GFP is 532 and 491 nm, respectively. Under bright-field, observation of the germline in live worms often is obstructed by other internal organs extending alongside, mainly the gut (Figure 3c). Furthermore, since the gut cells often generate autofluorescence signals, observation of fluorescence from germline nuclei is often perturbed when the gut is oriented in between gonad and the objectives. In LSFM images, individual germline nuclei, as well as nuclei in developing embryos, can be clearly distinguished even when the worm is placed in such orientation (Figure 3b, d, and 4b, e). A bright-field image of the live *C. elegans* is shown in Figure 4a. High-contrast embryo images obtained from the meta-lens LSFM are shown in Figure 4b, while a wide-field fluorescence image is shown in Figure 4c. Figure 4d–f shows corresponding zoom-in images to the dashed-box regions shown in Figure 4a–c. Therefore, the LSFM with the meta-lens can efficiently provide proper illumination for optical sectioning. Even with low magnitude objectives (20×, NA: 0.5), the nucleus of ∼8 µm in diameter from an individual oocyte can be clearly identified (Figure 3d). The results also demonstrate that the system shows a resolution in single-cell scale for *C. elegans*. The intensity cross-section profiles, along the lines plotted in Figure 3b and c, are shown in Figure 3e. The intensity profile from the bright field image demonstrates no specific features. However, the intensity profile from the LSFM shows a clear profile for the oocyte. This comparison confirms the fine capability of our system to observe *in vivo* images of *C. elegans* with cellular resolution in real-time. Moreover, as shown in Figure 4b, individual nuclei in late stage-embryos in the *C. elegans* can be clearly distinguished in the LSFM image.

**Figure 5:**
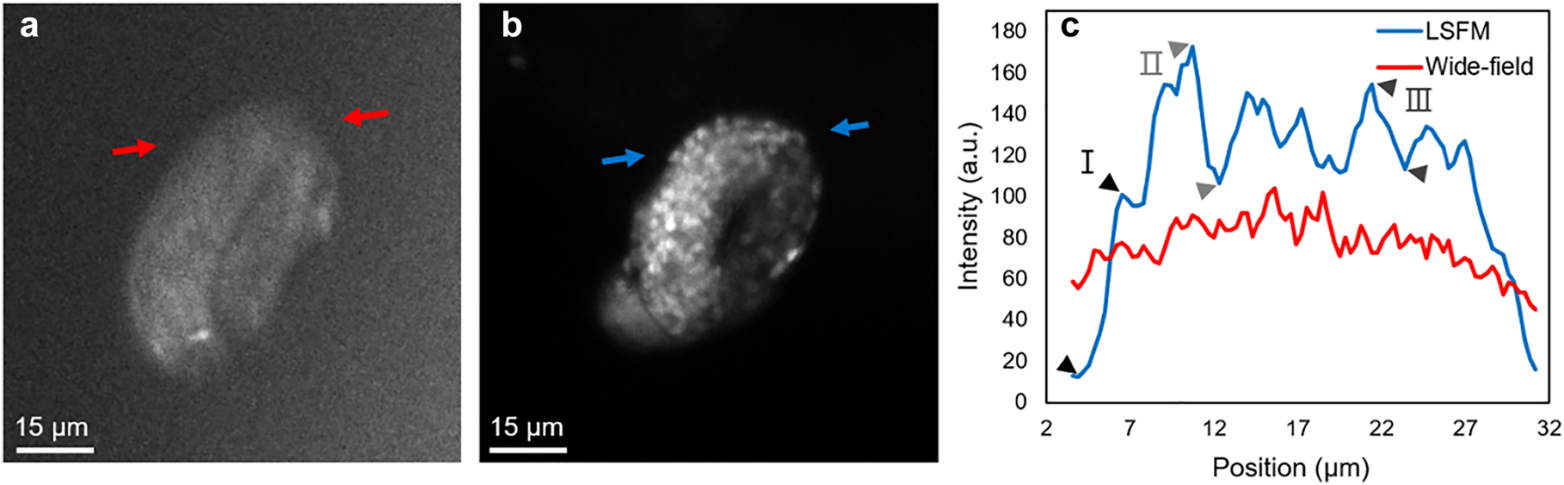
*In vivo* fluorescence images of developing embryos. (a) Wide-field fluorescence image of the embryo. (b) LSFM image of the embryo immobilized in agar. (c) Intensity cross-sections along the lines plotted in (a) and (b), respectively. I, II, and III indicate three different locations inside the embryos with a contrast value of 77, 24, and 15% using the meta-lens-equipped LSFM, while the corresponding value using the wide-field fluorescence microscopy is 14, 6, and 6%, respectively. (a) and (b) are shown in the same scale (time-lapse video can be found in supplementary materials).

In addition, to investigate optical sectioning capability in a moving object, we have examined our system to observe late stage *C. elegans* embryos, which exhibit constant twitching and rotation inside the egg, as shown in Figure 5. A wide-field fluorescence image of the late-stage embryo (Figure 5a) shows low contrast and poor distinction among individual nuclei. Contrarily, the LSFM with the meta-lens captures embryo movement with decent contrast in Figure 5b. Figure 5c shows intensity cross-sections comparing the signal-to-background with wide-field fluorescence microscopy and our LSFM system. Time-lapse animation of imaging *in vivo* late stage *C. elegans* embryos can be found in Supplementary video 3. Changes in structures of constant twitching and rotation over a period of ∼200 s can be clearly observed. Taken together, the experimental results further confirm the capability of our meta-lens-equipped LSFM system for multicolor imaging applications, which is of fundamental interest for various biomedical research fields [42]. Notably, our meta-lens-equipped LSFM system shows comparable image capability with conventional LSFM, which uses a combination of an objective and a conventional cylindrical lens as the illumination arm (Figure S8). Compared to the length of the illumination arm used in the conventional LSFM, the thickness of the meta-lens is significantly thinner. Therefore, the whole LSFM system can be dramatically miniaturized while maintaining a similar imaging capability for the targeted biospecimens.

In this work, we demonstrated a simple and effective use of nanophotonic meta-lens for realizing a compact light-sheet microscope for biomedical imaging. By adapting nanophotonic metasurface optics into a microscope, the system size can be effectively reduced while the imaging capability for the targeted biospecimens still remains. To the best of our knowledge, this is the first report on the fluorescence imaging of live *C. elegans* by light-sheet microscopy. With the well-designed meta-lens, the illumination arm of our LSFM reduced significantly, and the system shows a similar resolution of the cellular structures as the conventional counterpart. Unlike conventional refractive lenses, light-sheets parameters can be effectively tuned during the design process for increasing functionalities of LSFM. One of the major issues with any LSFM setup is the sample space between the two high NA objectives used in the detection, as well as illumination. As the working distance decreases with increasing the NA of objectives in both detection and illumination, which limits space for samples as well as scanning ranges [46]. In this context, reducing the complexity of the illumination will greatly relax such limitations. Further, the compact size and planar geometry of meta-lens allow its integration even with a sample holder to make a compact illuminator for LSFM. The impact of current work will be significantly visible: (a) in designing a portable and miniature microscope, (b) by adapting meta-lens in commercial LSFM and other advanced setups. For any futuristic miniature LSFM setup that includes microfluidics channels for lab-on-chip applications, fiber optics, or endoscopic probes, manipulation of light with metasurface-based optical element will outperform any other conventional refractive/diffractive optical components due to their inherent unique properties. The live imaging of the worms will facilitate a deep understanding of the intricate developmental biological process in *C. elegans*, which will be of great importance to developmental and medical applications. The reported system is inherently associated with all the advantages of LSFM, which will directly help in performing developmental biological studies for bio-specimens in real-time for longer durations. At present, the meta-lens is used only for illumination; however, with further experimentation, the LSFM system, which uses meta-lenses for both the detection and excitation, can be realized. The techniques discussed in this work for reducing the instrument size are not only beneficial for research-level microscopy but also compact microscopes for teaching and industrial purposes, such as multihead microscopes for parallelizing imaging.

## 3 Conclusions

In conclusion, we have shown the implementation of a metasurface optical element in LSFM for imaging live biological samples. The performance of our LSFM was evaluated by utilizing standard fluorescent microspheres and *in vivo* imaging of fine structures associated with the developmental process in live *C. elegans*. Results obtained from our systems clearly show internal structures of the *C. elegans* up to cellular resolution for multiple wavelengths. Experimental measurements are compared with the conventional LSFM systems (i.e., without any meta-lenses), and similar image resolution is demonstrated. The results clearly illustrate the capability of the system for biomedical imaging applications. Moreover, the significance of the present techniques lies in the fact that the complexity of the instrument in the illumination is entirely reduced, from tens of centimeters to 800 nm, without affecting any imaging performance, which clearly shows great potentials to utilize metasurface optics for miniaturizing microscopic imaging instruments. Applications of meta-lens can be readily extended to illumination, as well as in the detection of LSFM to include all the essential features of metasurfaces. Metasurface optics can be utilized for many LSFM modalities to achieve novel functionalities, such as meta-lens arrays [10] for light-sheet generation, multi-view/multi-dimensional selective plane illumination microscopy [47, 48], near-infrared [49] and multi-photon LSFM [50]. Further, by integrating beam shaping techniques in the meta-lens design, exotic light-sheet patterns with extended field of view and smaller thickness can be achieved. We believe the present work is an advancement of the application areas of metasurface in the field of microscopy.

## 4 Methods

### 4.1 Experimental setup for the meta-lens-equipped LSFM

**Figure 6:**
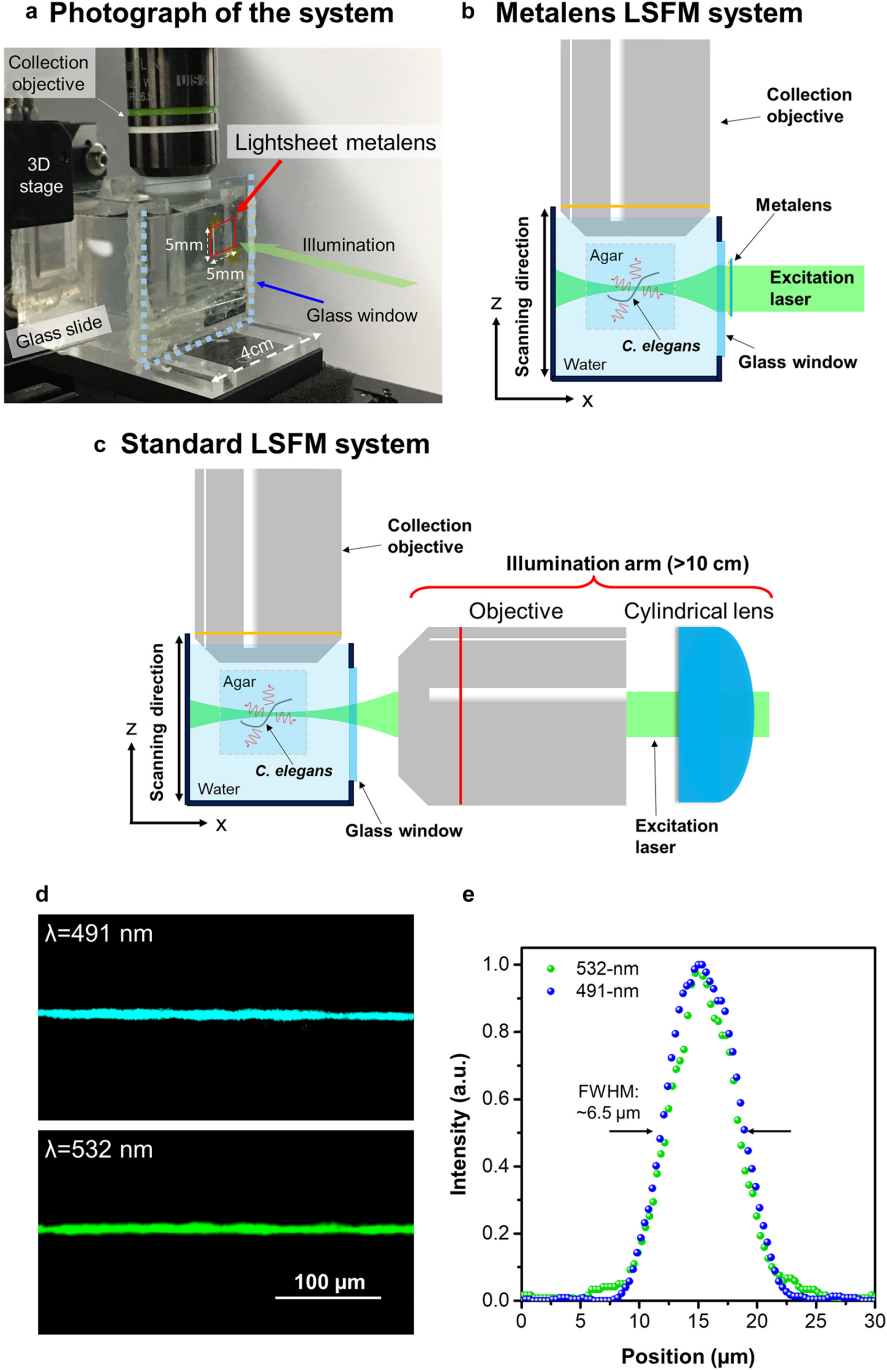
Meta-lens-equipped LSFM. (a) Photograph of the LSFM system. (b) Schematic of the experimental setup for the meta-lens-equipped LSFM. (c) Schematic of the setup for the standard LSFM. (d) Cross-sectional views of the light-sheets generated in water along the y-z plane at 491 nm (top panel) and 532 nm (bottom panel), respectively. (e) Corresponding intensity profiles of the respective light-sheet at different wavelengths along the *z*-axis.

To realize the ultracompact LSFM system, we designed and built an experimental setup that integrates the meta-lens for illumination (Figure 6). A photograph, as well as a schematic of it, is shown in Figure 6a and b, respectively. To characterize the performance of the light-sheet meta-lens, the intensity profiles of the generated light-sheet along the optical axis were measured (Figure S6). The substrate of the light-sheet meta-lens was cut into a 5 mm × 5 mm square and taped on a microscope glass slide as the illumination component. It was mounted on a three-dimensional stage to precisely control the illumination condition for the *C. elegans*. Due to the ultracompact size of the meta-lens, it enables the system to have design flexibility to create more sample space. An acrylic sample holder with a thin glass window was designed and made. The sample holder can well fit in the space between the meta-lens and the detection objective, and it was placed on a piezo stage for axial scanning. In this way, this system was able to precisely image different layers of the samples. The light-sheet meta-lens was placed adjacent to the glass window of a sample holder. To monitor the fluorescent images from the illuminated section of the sample, a water immersion objective (UMPLAN FLN 20×, NA 0.5) and an sCMOS camera (Hamamatsu ORCA4.0) were used. The excitation laser was incident from the substrate of the light-sheet meta-lens, and light-sheet illumination was generated in the sample holder to excite fluorescence within the tagged *C elegans*. A long-pass filter was placed between the camera and the objective detection lens to filter out the excitation. With the light-sheet meta-lens, the size of the illumination part is less than 1 × 1 mm, and 800 nm in thickness. Notably, in general, the illumination arm in a standard LSFM is of few tens of centimeters in length [12]; therefore, with the light-sheet meta-lens, the size of illumination is reduced by more than six orders of magnitude. The cross-section views of the light-sheets with different laser wavelengths are shown in Figure 6d. The light-sheets with uniform intensity distributions in water can be observed in the images. The full width at half maxima (FWHMs) of the light-sheets for 532-nm and 491-nm are around 6.5 µm (Figure 6e). The results show that the LSFM system shows a much compact system size compared to the conventional counterparts, and it is capable of producing light-sheets at different excitation wavelengths.

In the experimental measurements, the light-sheet meta-lens was taped on a cleaned glass slide and mounted on a three-dimensional linear stage (3D stage) to precisely control the light-sheet position. The sample was placed in water in the sample holder placed on another 3D stage for sample tracing and scanning. The incident laser was illuminated from the backside of the light-sheet meta-lens. A water immersion objective was used to collect the fluorescence from the illumination section of the live *C. elegans*. A long pass filter was placed between the objective and the sCMOS camera to filter out the excitation signal.

## Supplementary Material

Supplementary Material Details
