# Supplementary material for: Meta-lens light-sheet fluorescence microscopy for in vivo imaging
Source: Nanophotonics. 2022 Feb 21;11(9):1949–59. doi: 10.1515/nanoph-2021-0748 (PMC11501894; doi:10.1515/nanoph-2021-0748)
Supplement: Supplementary file 1 — Supplementary Material Details [file j_nanoph-2021-0748_suppl.docx]

Yuan Luo,* Ming Lun Tseng, Sunil Vyas, Ting-Yu Hsieh, Jui-Ching Wu,* Shang-Yang Chen, Hsiao-Fang Peng, Vin-Cent Su, Tzu-Ting Huang, Hsin Yu Kuo, Cheng Hung Chu, Mu Ku Chen, Jia-Wern Chen, Yu-Chun Chen, Kuang-Yuh Huang, Chieh-Hsiung Kuan, Xu Shi, Hiroaki Misawa, Din Ping Tsai *

Supplemental Materials for

Meta-lens light-sheet fluorescence microscopy for in vivo imaging

Note S1. Design of the unit cells

The design and simulation of the optical properties of the GaN nanopillars were performed using commercial software CST Microwave Studio, based on ﬁnite-difference time-domain method (FDTD). In the simulation, periodic boundary conditions were applied along the x-axis and the y-axis. In Fig. S1, the incident light is normally incident on the unit cells from the substrate. The optical constants of GaN in the visible regime are accessed from Ref. [1] of the supplementary information. The phase modulation and the transmission of the GaN nanopillars can be simulated accordingly. Due to the high refractive index of GaN, well-designed GaN nanostructures can effectively harvest incident light and various photonic resonances can be excited in the nanostructures. Therefore, the interaction between the GaN nanostructure and the incident light can be strong, resulting in a very significant modulation for the output light. An additional advantage for using GaN for metasurfaces is associated with its extremely wear resistance [2]. This unique property makes it very suitable for durable and stable nanophotonic devices. The near-filed distributions of the GaN nanopillars with different diameters at 532nm are demonstrated in Fig. S1. As can be seen in the simulation results, different waveguide-like cavity resonances are excited in the GaN nanopillars, which can effectively modulate the phase of the output light wave.


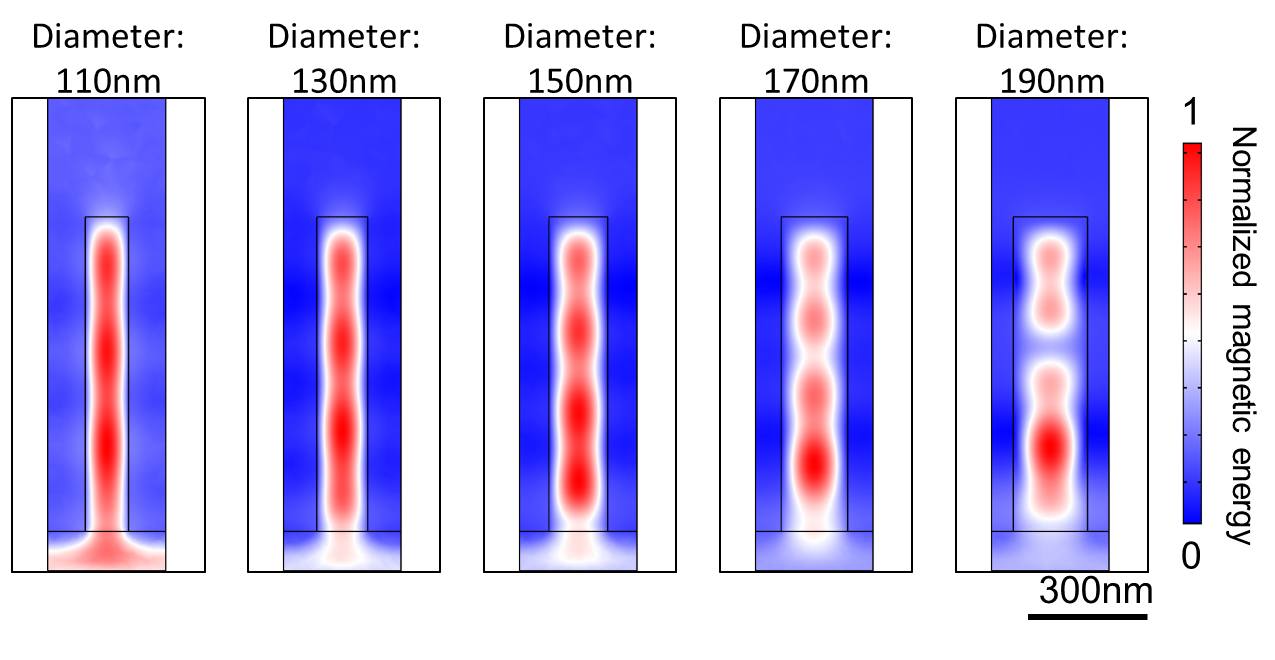


Fig. S1: Normalized magnetic energy of nanopillars as the subwavelength nanoresonator in the light-sheet meta-lens.

Note S2. Phase profile of light-sheet meta-lens

The design of the computer generated phase mask is the first step towards the realization of the meta-lens in the physical form. Utilizing the capability of metasurface to modulate the phase delay spanning the entire 2π phase over the broadband range, here, we first design the phase mask for cylindrical lens under thin lens approximation. The phase function of the lens is given by,

$\boldsymbol{\varphi}_{\boldsymbol{CML}}=\frac{\mathbf{2}\boldsymbol{\pi}}{\boldsymbol{\lambda}}\left( \sqrt{\boldsymbol{x}^{\mathbf{2}}+\boldsymbol{f}^{\mathbf{2}}}-\boldsymbol{f} \right)$ (1)

where, $\boldsymbol{\varphi}_{\boldsymbol{CML}}$ is the phase profile of light-sheet meta-lens, *f* is the focal length of the meta-lens, and λ is the wavelength of light used for illumination. In our case, we choose the design parameters according to the need of our biological specimen. The size of the aperture is D = 1 mm, focal length f = 10 mm, design wavelength λ = 532 nm and NA = 0.05. The result-ing phase profile for the light-sheet meta-lens is shown in Fig. S2.


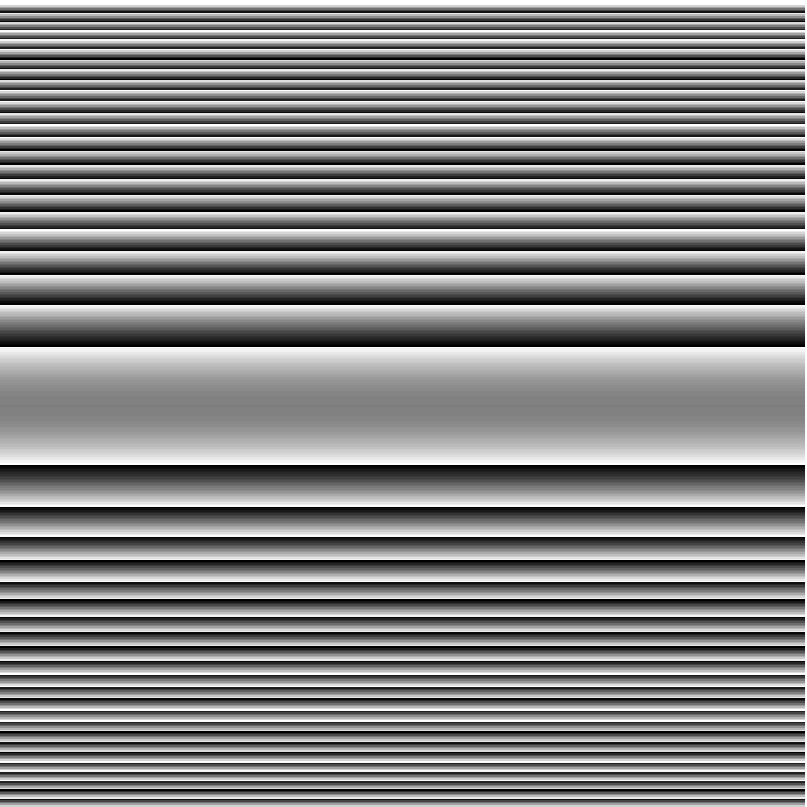


Fig. S2: Phase profile for designing light-sheet meta-lens

Note S3. Nanofabrication of the light-sheet meta-lens

The fabrication method for the light-sheet meta-lens is shown in Fig. S3. To fabricate the meta-lens, a 400-nm SiO_2_ layer was deposited an 800-nm GaN film using PECVD. A layer of ZEP-520A e-beam resist (thickness ∼ 100 nm) was spin-coated on the top of the sample. Another e-spacer layer was then coated on the sample before performing e-beam exposure to avoid the charging effect. Electron beam patterning was performed by using an E-Beam Writer System (Model: Elionix ELS-7000 at an acceleration voltage of 100kV with a beam current of 100 pA. After e-beam exposure, development was performed by immersing the substrate in a ZEP-N50 solution at room temperature for 60s. A 40-nm Cr layer was deposited on the sample by using an e-beam evaporator (base pressure: 5 × 10^‑7^ Torr; evaporation rate: 0.5 nm/s). The lift-off was performed by immersing the sample in N,N-Dimethylacetamide (ZDMAC) to prepare the mask for the following etching processes. The hard mask etching was carried out using the reactive ion etching method, while the etching of GaN was performed using an inductively-coupled-plasma reactive ion etching (ICP-RIE) system (mixture: BCl_3_/Cl_2_, ICP source power: 700 W, bias power: 280 W). Finally, the SiO_2_ mask on the top of the meta-lens was removed using a buffered oxide etch. An SEM image of the nanopillars in the light-sheet meta-lens is presented in Fig. S4, which confirms the fidelity of the nanofabrication to the original design.


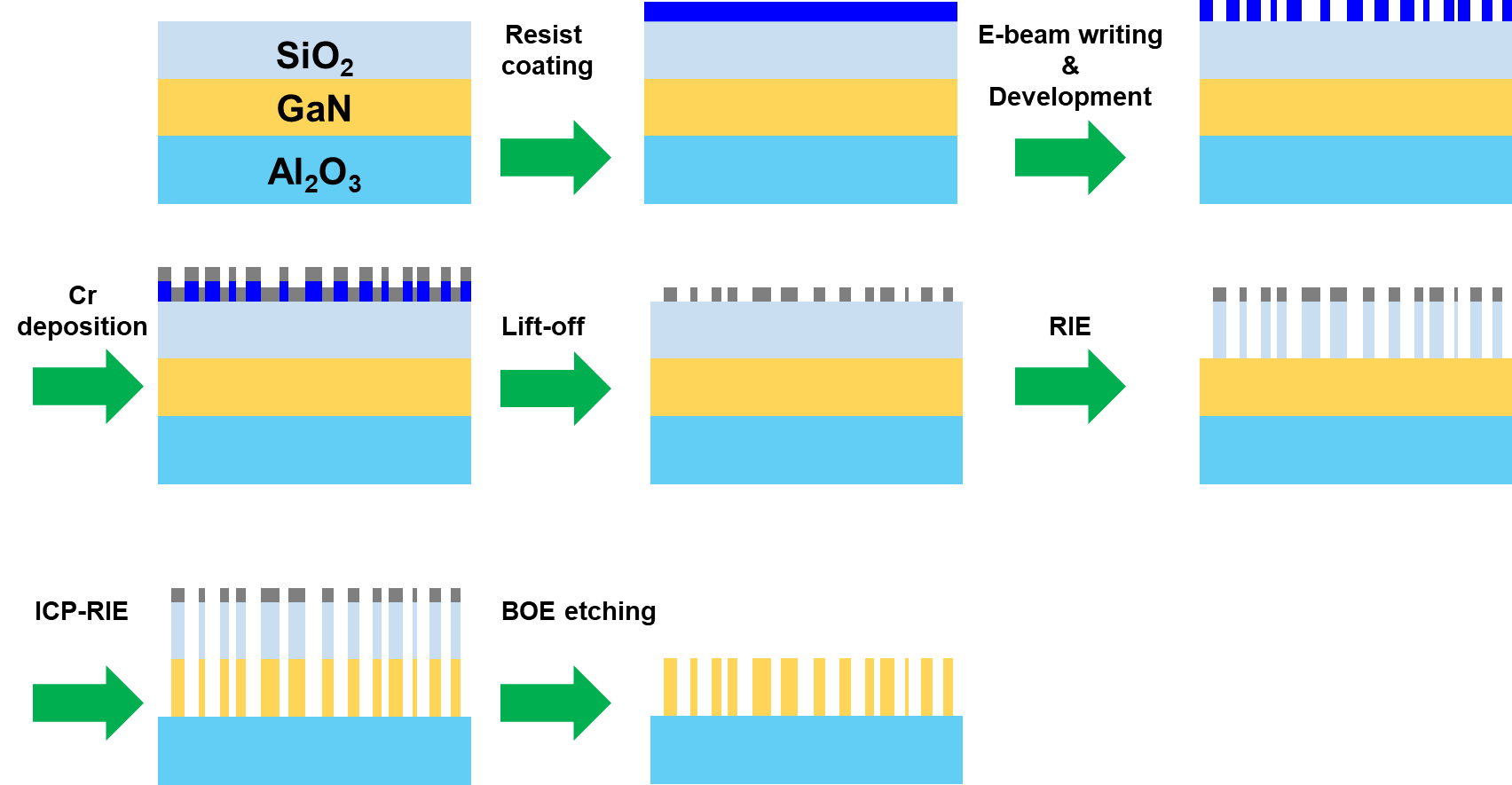


Fig. S3: Nanofabrication of the light-sheet meta-lens.

**
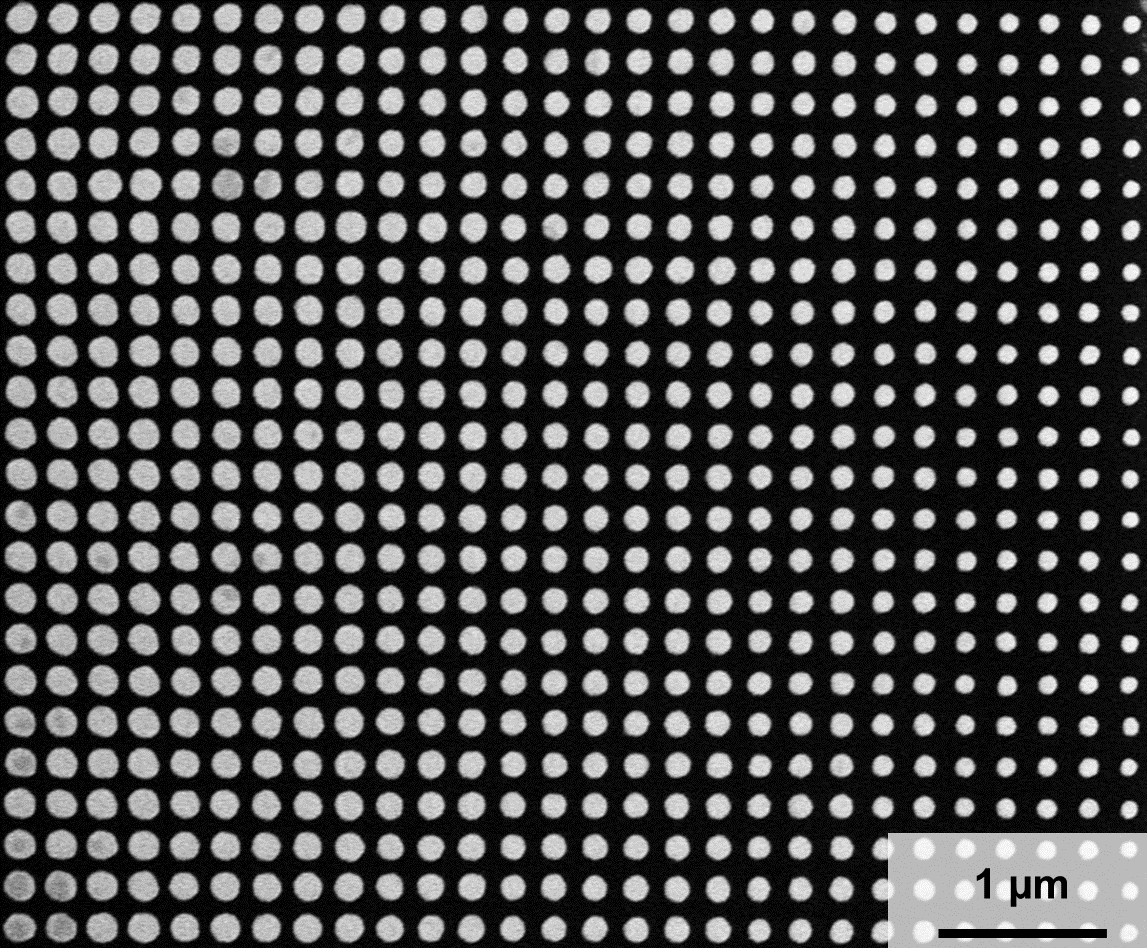
**

Fig. S4: Top-view SEM image of the nanopillars in the light-sheet meta-lens.

S4. Comparison of imaging capability

In the light sheet microscope, illumination and detection arms are separated, and the overall imaging quality is not only depending on the detection arms but also on the illumination arm. On the one hand, the lateral resolution is decided by the detection objective; the axial resolution of the system is decided by the thickness of the light sheet (optical sectioning capability). As long as the specification of the detection optics (including tube lens, microscope objective, and CCD pixel size) used in our setup is identical with any other conventional light-sheet microscope set up the resolution and hence the imaging quality will be identical. In the present case, we used a commercial microscope (Olympus Inc.) to show the adaptability of the meta-lens in any optical microscope. The lateral image resolution in our microscope is (0.62 μm), which exactly matches with the conventional light-sheet microscope designed with the identical optical components in our laboratory. Higher resolution can be achieved with the high NA objectives. However, there is a tradeoff between the high resolution and the field of view (FOV). Here, we show a specific example of in-vivo imaging of *C. elegans*. In our experiments, a biological sample *C. elegans* kept inside the agar was used. In order to minimize refractive index, mismatch a water immersion objective (UMPLAN FLN 20×, NA 0.5) is used. The lateral resolution of our setup is exactly equal to any other conventional light-sheet microscope setup of the similar detection optics specifications (including tube lens, microscope objective, and CCD pixel size). In order to further compare the imaging performance, we included the results of the conventional light-sheet microscope in the supplemental results as Fig. S5 (a). The theoretically calculated lateral resolution for a conventional light-sheet microscope is 0.61 μm. A comparison of LSFM images recorded by a conventional LSFM system with our meta-lens-equipped system is presented in Figure S5 (b). As can be seen, individual nuclei can be clearly observed in both fluorescence images. The results confirm that our system shows comparable image capability with a simple conventional LSFM system.


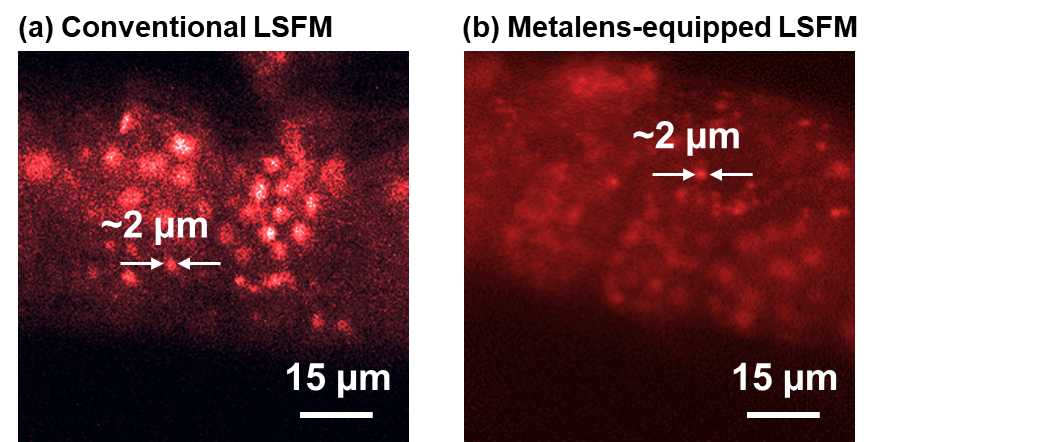


Fig. S5: Comparison of fluorescent images of C. elegans measured by using the (a) conventional LSFM system and (b) the meta-lens-equipped LSFM system.

S5. Characterization of the light-sheet meta-lens

To characterize the performance of the light-sheet meta-lens, the intensity profiles of the generated light-sheet along the optical axis were measured. In the measurement, a super-continuum laser system was used as the light-sheet. The wavelength was set to be 532-nm by using an Acousto-optic tunable filter (AOTF). The spot size was carefully adjusted to be around 1mm in diameter. The laser was incident from the substrate side of the meta-lens. An objective and a CCD were set on a motorized stage. To record the intensity profiles along the optical axis, the relative distance between the objective and the meta-lens was varied, and the profile was subsequently recorded (Fig. S6 and Supplementary video 2). More details can be found in our previous work [3]. At the operating wavelength of 532 nm, the average focusing efficiency of our designed meta-lens is 40%, which is defined as the ratio of the power of the produced beam to the power of incoming light

**
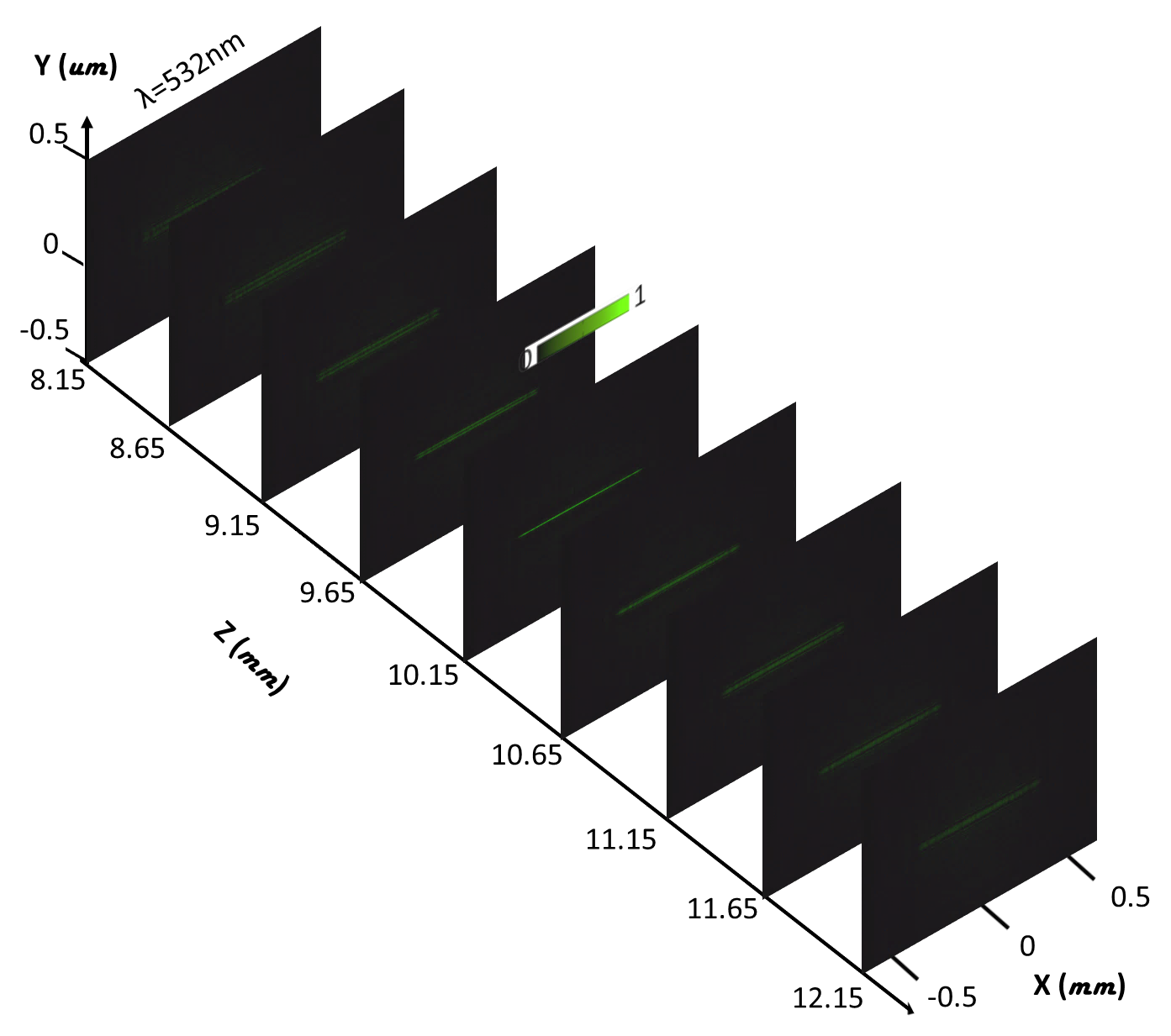
**

Fig. S6: Intensity profiles of the light-sheet along the optical axis (z-axis).

S6. Comparison of experimental setup between standard LSFM and meta-lens-equipped LSFM

Separated illumination and detection of sample in light-sheet microscopes provides unique advantages [4]. While the most important feature of the light-sheet microscope is its optical sectioning capability that originates from its illumination condition and it directly controls the overall imaging performance of the system, the collection of fluorescence images in a widefield manner makes it a more efficient and high-speed method as a comparison to other methods. Reducing complexity at any level of the illumination arm will greatly enhance its applicability and usage. With metasurface optics, not only the sizes of the optical components but also the overall size of the optical system can be significantly reduced. One common approach to generating a light-sheet for LSFM is shown in Fig. S7 (a). A cylindrical lens is used to shape the incident laser beam into a sheet-like form, and an adjacent objective subsequently shrinks the size of the light-sheet and focuses it on the sample. A photograph of the conventional illumination arm is shown in Fig. S8. The total length of the illumination arm is around 15 cm. It needs additional bulky steel holders to fix it, making the alignment and arrangement of the illumination arm much difficult. While the conventional illumination needs multiple components to realize the ideal light-sheet, a meta-lens can be designed to create a light-sheet with a submicron thickness. As shown in Fig. S7 (b) and Fig. 6 (in the main text), the light-sheet meta-lens is extremely compact and lightweight. Compared to the illumination arm in standard LSFM, light-sheet meta-lens demonstrates similar functionality but has much smaller thickens 8x10-5 cm (i.e., 800 nm) with a weight of less than half milligrams. Therefore, the light-sheet meta-lens provides a great platform for realizing a simple, compact, and lightweight LSFM system with high-resolution imaging capability. It contains several components for achieving the ideal illumination.


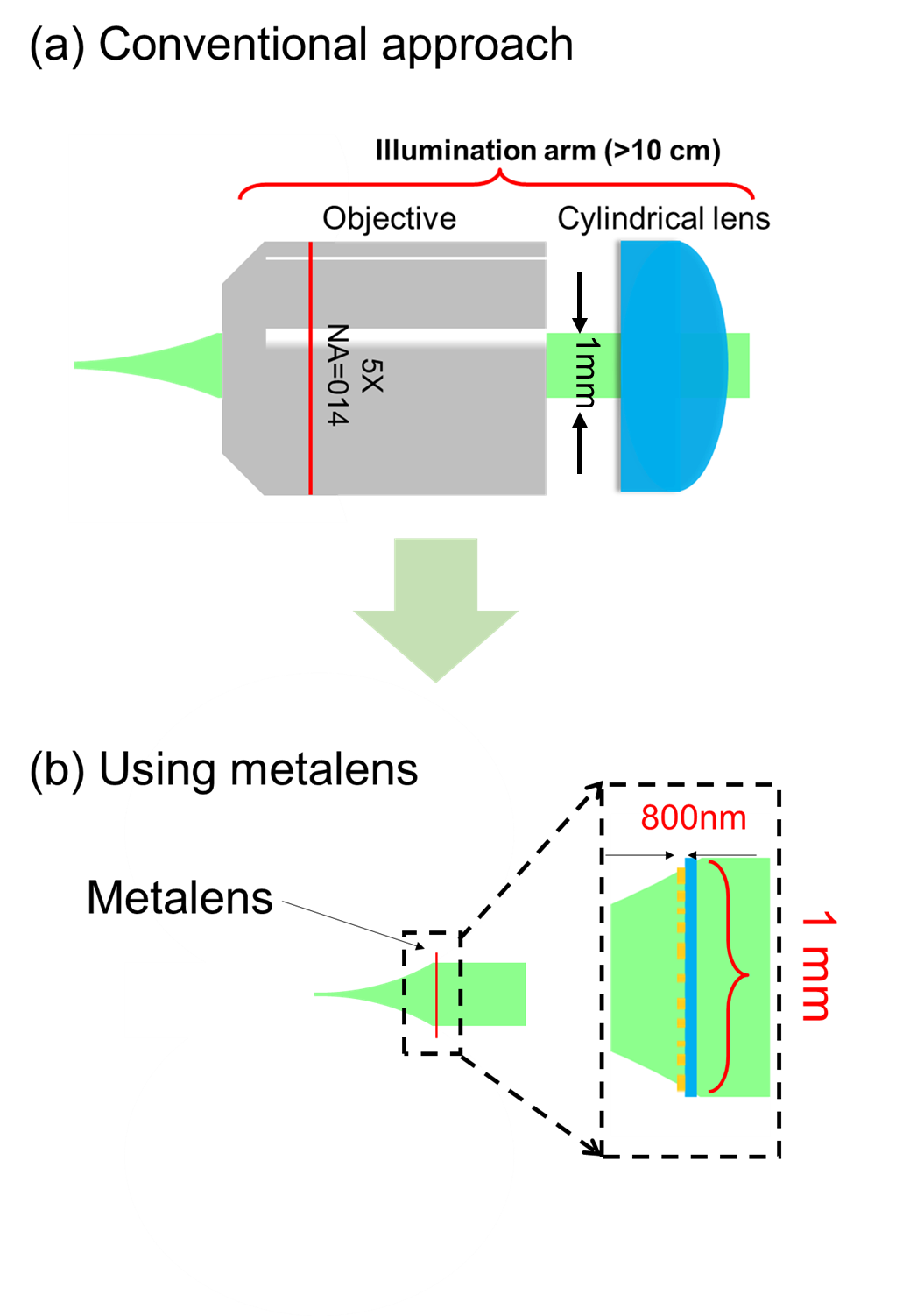


Fig. **S7**: Comparison of different approaches to generate a light-sheet. (a) the standard method and (b) using a light-sheet meta-lens to produce a light-sheet for LSFM. The reported meta-lens shows a similar functionality as the conventional illumination arm but has a much smaller device size.

**
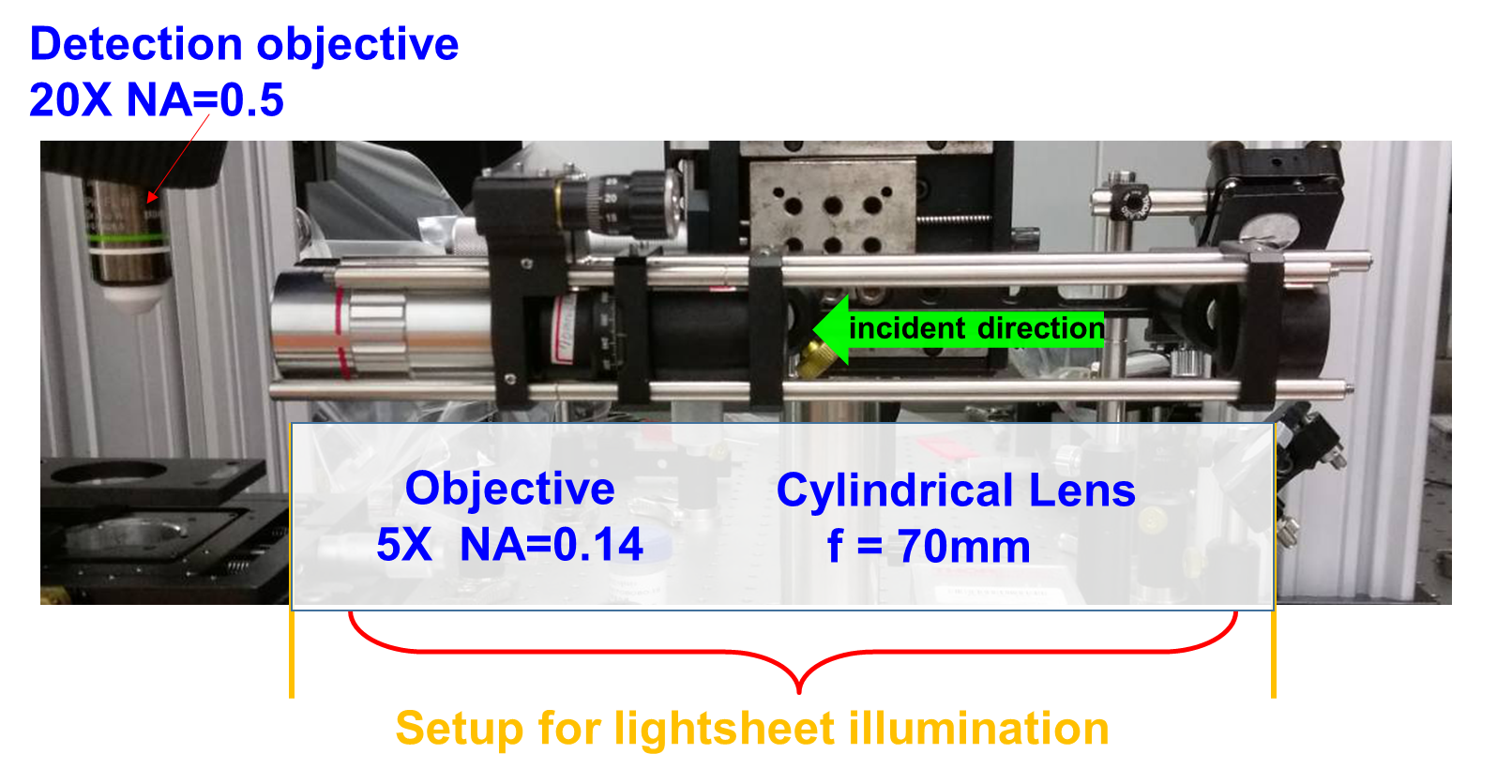
**

Fig. S8: Photograph of illumination arm for standard LSFM.

S7. Comparison between DOEs and metasurfaces

Most of the functions performed by the meta-lens can also be obtained with conventional diffractive optics elements (DOEs). However, the main difference appears when the size is in few microns. In general, conventional DOEs control incident light characteristics through micro-level structures. When the aperture size is small, the number of such micro-structure elements controlling light is limited that seriously hinders the modulation capability of conventional DOEs. On the other hand, nano-level structures (meta-atoms) inside the metasurface allow a high density of light-controlling units even within tiny apertures. This difference in the size and density of unit cells between these two techniques (e.g., conventional DOEs and metasurface) is at least three orders of magnitude, resulting in drastic changes in the resolution, quality of mode, and precise control of the amplitude, phase, and polarization properties of the output light field. Hence, the meta-lens will be more efficient, effective and provides unparalleled advantages over alternative techniques.

S8. Nematode strains and manipulation

The following strains of *C. elegans* [5] were obtained from the Caenorhabditis Genetics Center (CGC): OD56 *unc-119(ed3) III; ltIs37 [(pAA64) pie-1p::mCherry::his-58 + unc-119(+)*] IV, JJ1850 *unc-119(ed3) III*; *zuIs178 [his-72(1kb 5' UTR)::his-72::SRPVAT::GFP::his-72 (1KB 3' UTR) + 5.7 kb XbaI - HindIII unc-119(+)]*.

Reference

[1] A. S. Barker, and M. Ilegems, “Infrared Lattice Vibrations and Free-Electron Dispersion in GaN,” *Physical Review B,* Vol. 7, no 2, pp. 743-750, 1973.

[2] G. Zeng, C.-K. Tan, N. Tansu, and B. A. Krick, “Ultralow wear of gallium nitride,” *Appl. Phys. Lett*., Vol. 109, no 5, p. 051602, 2016.

[3] S. Wang, et al. “A broadband achromatic metalens in the visible,” *Nat. Nanotechnol.,* Vol. 13, no 3, pp. 227-232, 2018.

[4] O. E. Olarte, J. Andilla, E. J. Gualda, and P. Loza-Alvarez, “Light-sheet microscopy: a tutorial,” *Adv. Opt. Photon*., Vol. 10, no 1, pp. 111-179, 2018.

[5] T. Stiernagle, “WormBook: the online review of C. elegans biology,” *WormBook*, 1-11, 2006.
